# Amyloid-*β* reduces the expression of neuronal FAIM-L, thereby shifting the inflammatory response mediated by TNF*α* from neuronal protection to death

**DOI:** 10.1038/cddis.2015.6

**Published:** 2015-02-12

**Authors:** P Carriba, S Jimenez, V Navarro, I Moreno-Gonzalez, B Barneda-Zahonero, R S Moubarak, J Lopez-Soriano, A Gutierrez, J Vitorica, J X Comella

**Affiliations:** 1 Institut de Recerca de l'Hospital Universitari de la Vall d'Hebron (VHIR), Passeig Vall d'Hebron 119-129, Barcelona 08035, Spain; 2 Institut de Neurociències, Departament de Bioquímica i Biologia Molecular, Facultat de Medicina, Universitat Autònoma de Barcelona, Campus de Bellaterra (Edifici M), Bellaterra 08193, Spain; 3 Centro de Investigación Biomèdica en Red sobre Enfermedades Neurodegenerativas (CIBERNED), Spain; 4 Instituto de Biomedicina de Sevilla (IBIS), Hospital Universitario Virgen del Rocio, Consejo Superior de Investigaciones Cientificas Universidad de Sevilla, c/ Manuel Siurot s/n, Sevilla 41013, Spain; 5 Departamento Bioquimica y Biologia Molecular, Facultad de Farmacia, Universidad de Sevilla, Sevilla 41012, Spain; 6 Departamento de Biologia Celular, Genetica y Fisiologia. Facultad de Ciencias. IBIMA Universidad de Malaga, Malaga 29071, Spain

## Abstract

The brains of patients with Alzheimer's disease (AD) present elevated levels of tumor necrosis factor-*α* (TNF*α*), a cytokine that has a dual function in neuronal cells. On one hand, TNF*α* can activate neuronal apoptosis, and on the other hand, it can protect these cells against amyloid-*β* (A*β*) toxicity. Given the dual behavior of this molecule, there is some controversy regarding its contribution to the pathogenesis of AD. Here we examined the relevance of the long form of Fas apoptotic inhibitory molecule (FAIM) protein, FAIM-L, in regulating the dual function of TNF*α*. We detected that FAIM-L was reduced in the hippocampi of patients with AD. We also observed that the entorhinal and hippocampal cortex of a mouse model of AD (PS1_M146L_xAPP_751sl_) showed a reduction in this protein before the onset of neurodegeneration. Notably, cultured neurons treated with the cortical soluble fractions of these animals showed a decrease in endogenous FAIM-L, an effect that is mimicked by the treatment with A*β*-derived diffusible ligands (ADDLs). The reduction in the expression of FAIM-L is associated with the progression of the neurodegeneration by changing the inflammatory response mediated by TNF*α* in neurons. In this sense, we also demonstrate that the protection afforded by TNF*α* against A*β* toxicity ceases when endogenous FAIM-L is reduced by short hairpin RNA (shRNA) or by treatment with ADDLs. All together, these results support the notion that levels of FAIM-L contribute to determine the protective or deleterious effect of TNF*α* in neuronal cells.

Alzheimer's disease (AD), the most common neurodegenerative disease among the elderly, is characterized by synaptic and memory defects, neuroinflammation, and progressive neuronal death. As in other neurodegenerative diseases, apoptosis is the main mechanism by which neurons die.^1, 2, 3^ This process has been reported to result from and be reinforced by the neuroinflammatory environment.^4, 5^

The brains of AD patients show high tumor necrosis factor-*α* (TNF*α*) expression.^6, 7, 8, 9^ Despite the elevated number of reports implicating this cytokine in this disease, the role of TNF signaling in AD is controversial. It has been reported that TNF*α* protects neurons against amyloid-*β* (A*β*) toxicity both *in vitro*^10^ and *in vivo*,^11^ but it also contributes to exacerbating neurodegeneration.^12, 13^

TNF*α* plays a central role in inflammation and apoptosis. TNF receptor 1 (TNFR1), the main receptor for TNF*α*,^14^ can trigger two signaling pathways: survival or death. Survival through the activation of the nuclear factor-*κ*B (NF*κ*B) and/or FLIP-L (FLICE-like inhibitory protein, long isoform)-dependent mitogen-activated protein kinase/extracellular-signal-regulated kinase (MAPK/ERK) pathways^15^ or, under certain circumstances, it can also trigger cell death by the induction of the extrinsic apoptotic pathway.^16^ Indeed, TNF*α* has the capacity to kill neurons only when the NF*κ*B transcription factor pathway is blocked^17, 18^ or the macromolecular synthesis is inhibited.^19, 20^ These observations suggest that in neurons several regulatory proteins prevent the induction of apoptosis at various stages of TNF signaling.^21^ One such protein is the long form of Fas apoptotic inhibitory molecule (FAIM) protein (FAIM-L).

The *FAIM* gene gives rise to two isoforms, the short (S) and the long (L) form. FAIM-S is widely expressed in most cells and tissues.^22^ However, in the nervous system FAIM-S does not exert an anti-apoptotic function.^23^ FAIM-L is expressed exclusively in neurons, where it serves as an antagonist of death induced by TNFR1 and FAS.^23^

In this study, we found that FAIM-L expression is reduced in hippocampal samples from AD patients and also in a transgenic mouse model of the disease, PS1_M146L_xAPP_751sl_ (PS1xAPP). In primary cortical neurons, A*β* reduced the expression of FAIM-L, thus suggesting that the expression of this protein is associated with the progression of the disease. We also show that the TNF*α* protection against A*β* toxicity is suppressed when FAIM-L expression levels are low (by RNA interference (RNAi) or by treatment with A*β*). Therefore, the response of neurons to the same inflammatory stimulus (TNF*α*) varies depending on the quantity of FAIM-L in the neuron. Thus, we provide a molecular basis through which to explain the protective or deleterious effect of TNF*α* in neuronal cells during the progression of AD.

## Results

### FAIM-L is reduced in hippocampal samples from AD patients and in the entorhinal and hippocampal cortex in the transgenic PS1xAPP mouse

The pathogenesis of AD involves multiple factors. In this regard, there are several lines of evidence indicating that TNF*α* signaling makes a considerable contribution to this disease.^24^ Here we used quantitative PCR (qPCR) to systematically analyze in postmortem hippocampal samples from AD patients the proteins implicated in this signaling pathway, including the antagonists of DRs: CASP8 and FADD-like apoptosis regulator (CFLAR, aliases cFLIP-L), Lifeguard, and FAIM-L (Supplementary Information and Supplementary Figure S1).

**Figure 1:**
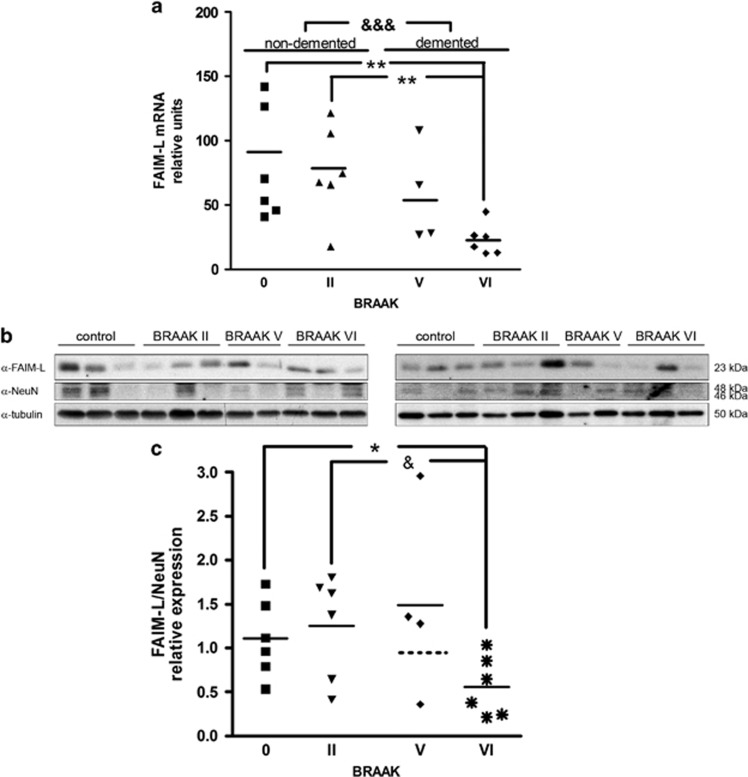
Decrease in FAIM-L expression in hippocampal samples from AD patients. (**a**) mRNA levels of FAIM-L. Statistical analyses were done with Kruskal–Wallis test followed by Dunn's multiple comparison test. ^&&&^*P*<0.0001 for comparisons between control nondemented (BRAAK 0 and BRAAK II) *versus* AD demented (BRAAK V and BRAAK VI); and **P<0.001 between the different stages. (**b**) Representative western blot of FAIM-L, NeuN was used for normalization and tubulin for loading control. (**c**) FAIM-L protein expression normalized with the neuronal marker NeuN, thereby discarding the possibility that the reduction was caused by neuronal loss. Mann–Whitney nonparametric *t*-test. *P=0.0382 BRAAK 0 *versus* BRAAK VI and ^&^*P*=0.0305 BRAAK II *versus* BRAAK VI. In both cases, data are mean±S.D. of three independent experiments

Among the proteins analyzed, FAIM-L was most clearly altered during the progression of BRAAK stages. BRAAK staging describes the amount and distribution of neurofibrillary tangles (NFT).^25^ This postmortem analysis is widely used because it has been found to correlate well with the severity of dementia.^26, 27, 28^ As FAIM-L is expressed only in neurons and has been described as an antagonist of TNF*α*-induced death,^23^ we focused on the role of this protein in AD. A notable decrease in FAIM-L mRNA was observed with the progression of the disease, being significant between control individuals (BRAAK 0 and BRAAK II) and demented AD patients (BRAAK V and BRAAK VI) (Figure 1a). Compared with nondemented patients, FAIM-L mRNA at BRAAK V decreased, although not reaching statistical significance. At BRAAK stage VI, FAIM-L mRNA was reduced to 30% of BRAAK 0 (individuals with no histopathological alteration of the brain but younger) or BRAAK II (asymptomatic subjects with similar age and with some histopathological modifications) (Figure 1a). Protein quantification of FAIM-L using the neuronal marker NeuN for the normalization revealed similar trend. Using NeuN allows discarding that the reduction of FAIM-L was due to neuronal death. In BRAAK V, we observed a diminution of FAIM-L levels but we were not able to detect significant difference, as there was one out of range sample. Nevertheless, at BRAAK stage VI, FAIM-L quantification showed a reduction of 50% compared with normal cognition individuals (BRAAK 0 or BRAAK II), confirming the results obtained by mRNA analysis (Figures 1b and c).

**Figure 2:**
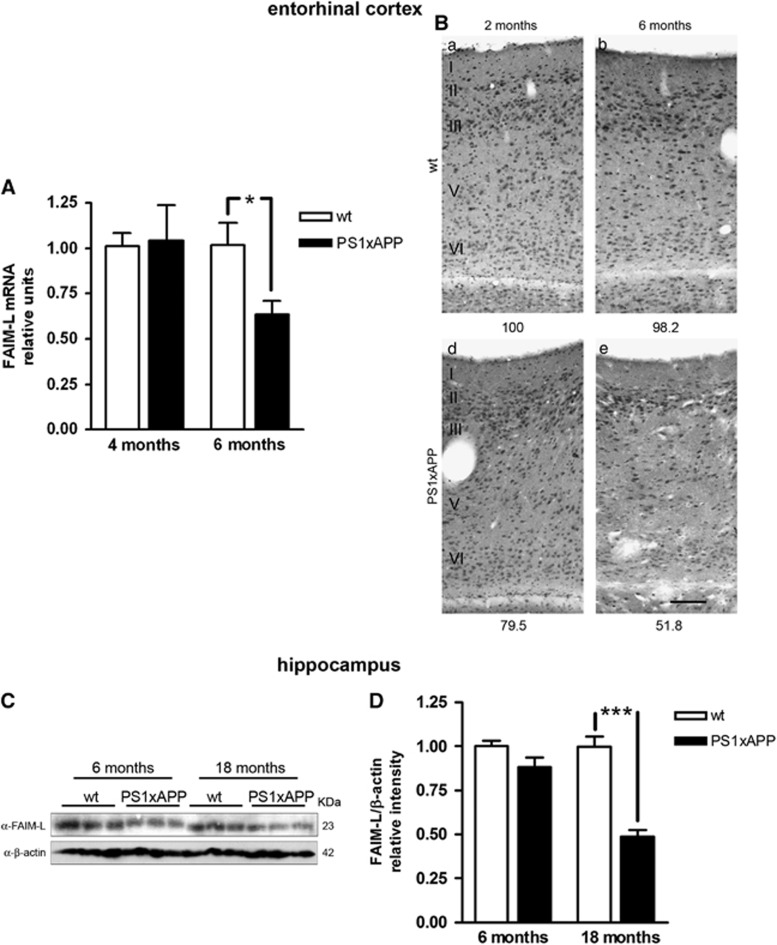
Reduction of FAIM-L expression in transgenic PS1xAPP animals. (**A**) FAIM-L mRNA levels in laser-microdissected entorhinal cortex. *T-test with *P*=0.039. Data are mean±S.D. of three independent experiments. (**B**) Immunodetection of FAIM-L expression in the entorhinal cortex. In the entorhinal cortex, WT animals display a similar immunostaining pattern for FAIM-L antibody at the ages analyzed (2 and 6 months old), without clear differences between superficial (I–III) and deep (IV–VI) layers. The PS1xAPP transgenic animals, however, present a marked reduction in FAIM-L labeling, slightly at 2 months of age, but patent at 6 months. The decline in FAIM-L immunoreactivity does not follow any apparent pattern, showing a reduction in all the layers, possibly less pronounced in neurons in layers II–III. Numbers at the bottom of the micrographs indicate the relative integrated density of the FAIM-L labeling. Scale bar,100 *μ*m. (**C**) Western blot of FAIM-L in samples from WT and transgenic animals at different ages. Three different animals were used to obtain homogenates samples for each condition analyzed. (**D**) Protein FAIM-L quantification in hippocampus using *β*-actin as a loading control. Data are mean±S.D. of three independent western blots with three animal samples for condition. *T*-test ***P<0.0001

The results in human samples prompted us to perform similar analysis in the AD transgenic mouse model PS1xAPP. These animals reproduce the temporal and regional neurodegeneration and neuroinflammation that occur in the brains of AD patients. At 6 months of age, these animals show degeneration in principal neurons and somatostatin/neuropeptide Y (SOM/NPY) interneurons in the entorhinal cortex.^29^ At this age, the analysis by qPCR in microdissected entorhinal cortex showed a significant reduction of FAIM-L mRNA in the transgenic animals compared with wild-type (WT) mice (Figure 2A). In addition, the immunodetection of FAIM-L in this cortical region displayed a marked reduction with age in the transgenic animal (Figure 2B).

In the hippocampus, PS1xAPP mice develop pyramidal neurodegeneration at ∼17–18 months of age.^30, 31, 32^ Western blot quantification of the expression of FAIM-L in the hippocampus of these animals revealed a noteworthy decrease at 18 months (Figures 2C and D). Thus, in both brain regions the decrease in FAIM-L expression occurs before the increase in neuronal death.^29, 32^

### A*β* reduces the expression of FAIM-L

**Figure 3:**
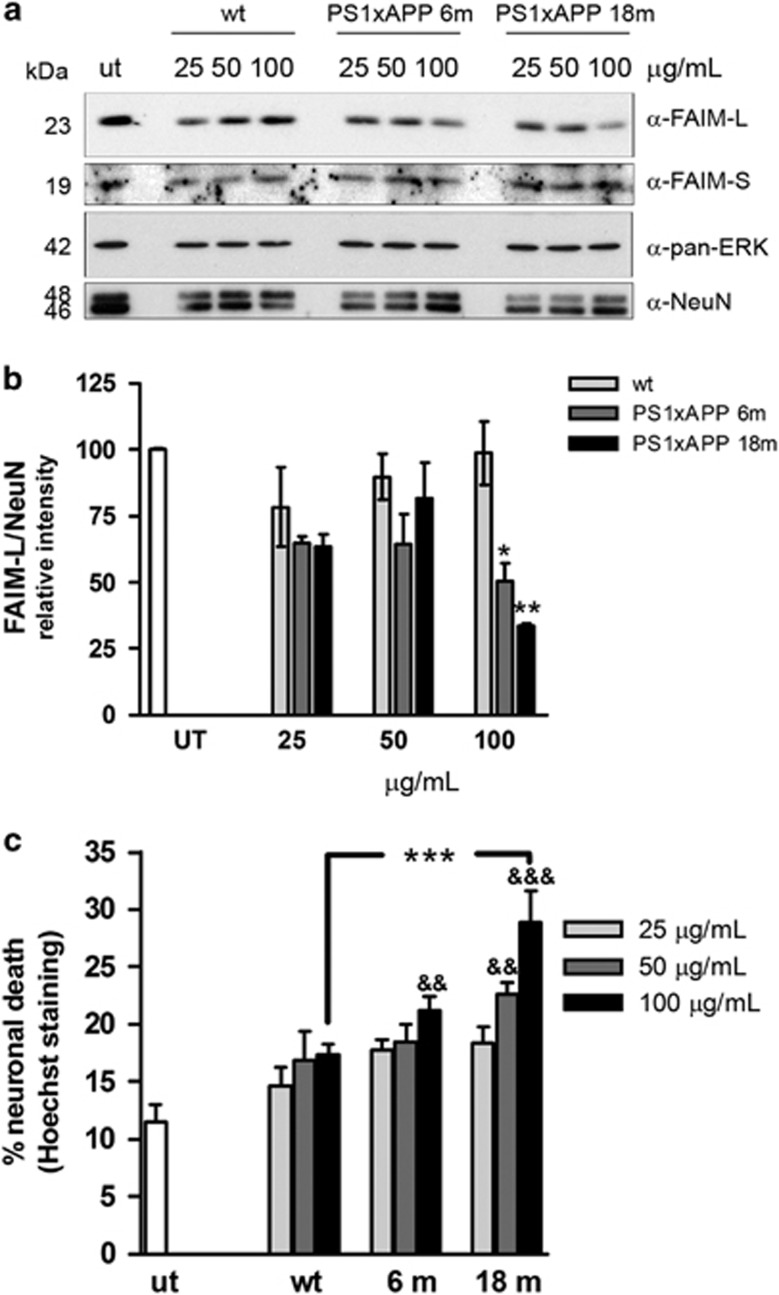
Cortical soluble fractions from the PS1xAPP mice reduce FAIM-L levels and also induce death in primary cortical neurons. (**a**) Primary cortical neurons were treated for 48 h with different amounts of soluble fractions from WT or 6- or 18-month-old transgenic mice. FAIM-L and FAIM-S were determined by western blot, using pan-ERK and NeuN as a control of equal loading. (**b**) Quantification of three independent western blots (**P<0.01 and *P<0.05, *t*-test). (**c**) Neurons were treated for 48 h with the indicated amounts of the soluble fraction from WT and PS1xAPP mice of different ages, and the neuronal death induced was determined by counting nuclei after Hoechst staining. One-way ANOVA with multiple Newman–Keuls test gives ***P<0.001 between WT and 18 months with 100 *μ*g/ml; and ^&&&^*P*<0.001 and ^&&^*P*<0.01 in relation to untreated neurons

In order to analyze the factors affecting the FAIM-L expression, we treated primary mice cortical neurons with soluble fractions from the cortex of PS1xAPP animals of different ages. By western blot, we observed a dose-dependent reduction of FAIM-L, but not FAIM-S, in neurons treated with the soluble fractions of the transgenic animals but not those treated with the soluble fractions of WT animals (Figure 3a). This reduction was significant for the soluble fractions from both 6- and 18-month-old animals at a final protein concentration of 100 *μ*g/ml in the culture media (Figure 3b). Thus, our data further confirmed *in vitro* what we have already observed in AD patients and in an AD animal model. We have previously reported the presence of oligomeric A*β* (oA*β*) in the soluble fractions of these transgenic animals.^32^ According to this, in cultured neurons these soluble fractions also induced death (Figure 3c). Nonetheless, the death of neurons did not explain the decrease in FAIM-L levels as FAIM-L protein quantification was corrected with the neuron-specific marker NeuN (Figures 3a and b).

**Figure 4:**
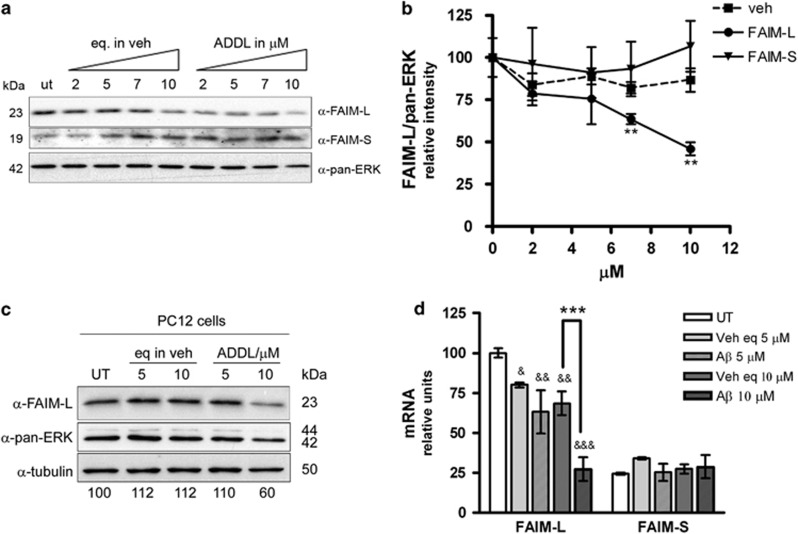
Amyloid-*β* reduces FAIM-L levels. (**a**) Primary cortical neurons were treated with the indicated amount of ADDLs and the corresponding vehicle control for 48 h and then processed for FAIM-L and FAIM-S immunoblotting. Pan-ERK was used as a loading control. (**b**) Western blot quantification of three independent experiments (**P<0.01, *t*-test). (**c**) PC12 cells were treated for 36 h with the indicated amount of ADDLs and the corresponding vehicle. FAIM-L immunoblot using pan-ERK and tubulin as a loading control. No significant differences were observed with both loading controls. Numbers at the bottom of the lanes indicate the amount of FAIM-L in relation to tubulin. (**d**) The qPCR for FAIM-L and FAIM-S after 48 h of the indicated treatments. ***P<0.001 between vehicle and 10 *μ*M ADDLs; and ^&&&^*P*<0.001, ^&&^*P*<0.01, and ^&^*P*<0.05 for comparisons with untreated neurons. One-way ANOVA with multiple Newman–Keuls test

The hippocampi of PS1xAPP mice show an increase in soluble oA*β* with age, specially the low-n oligomers.^32^ Therefore we questioned whether oA*β* causes the reduction in FAIM-L expression. To address this point, we treated primary neurons with increasing amounts of A*β*-derived diffusible ligands (ADDLs) and examined FAIM-L expression by western blot. The expression of FAIM-L, but not that of FAIM-S, in cultured neurons decreased in a dose-dependent manner in response to increasing concentrations of ADDLs (Figures 4a and b). Moreover, the decrease in endogenous FAIM-L in response to ADDL treatment also occurred in the neuron-like cell model PC12 (Figure 4c). As in human samples and the PS1xAPP mice, the downregulation of FAIM-L was also detected at the mRNA level in the cultured neurons (Figure 4d), thus suggesting that A*β* is modulating the expression of this protein rather than its degradation.

### FAIM-L does not modify A*β* toxicity

**Figure 5:**
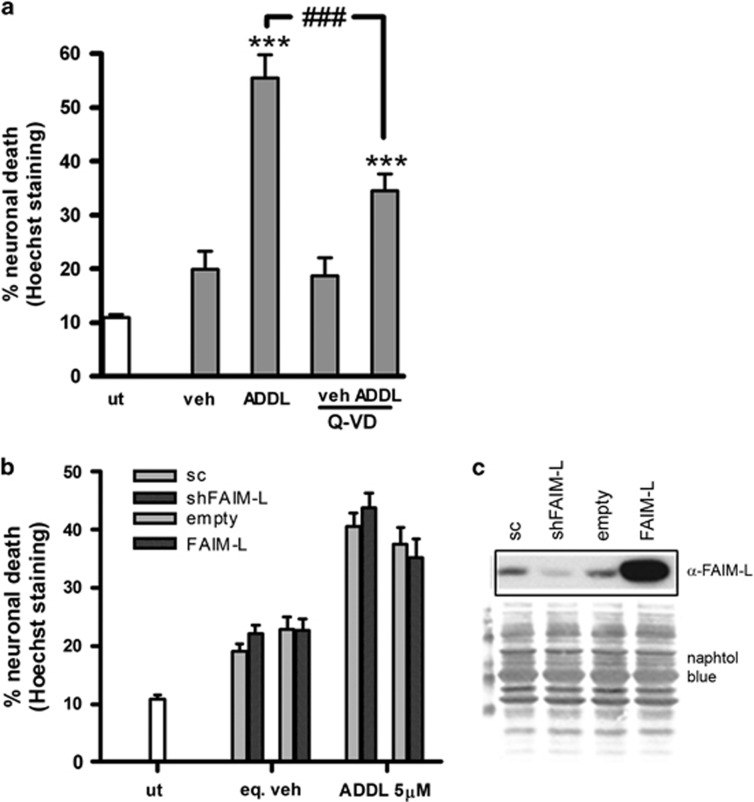
FAIM-L does not modulate A*β* toxicity. (**a**) Primary cortical neurons were treated with 10 *μ*M of ADDL or the equivalent in vehicle in the presence or not of 40 *μ*M of Q-VD for 48 h, and cell death was determined by counts of nuclear Hoechst staining. Data are mean±S.E.M. of three independent experiments. One-way ANOVA and Newman–Keuls *post hoc*. ***P<0.001 for comparison between untreated and vehicle-treated cells; ^###^*P*<0.001 ADDLs *versus* ADDLs+Q-VD. (**b**) Cortical neurons were transduced for 6 days with lentivirus (Lv) for downregulation (shFAIM-L) or overexpression (FAIM-L) of FAIM-L with the corresponding scrambled (sc) or empty controls. Cells were then treated with 5 *μ*M ADDLs or the equivalent in vehicle for 48 h. Neuronal death was measured by counting nuclei stained with Hoechst. Data are mean±S.E.M. of three independent experiments. (**c**) FAIM-L immunoblot after infection. Loading control determined by Naphthol blue

FAIM-L is a potent antagonist of death induced by the activation of the death receptors (DRs) TNFR1 and FAS.^23^ DRs are cell surface receptors that belong to the TNF superfamily and that trigger apoptosis upon ligand binding. There are conflicting reports regarding the mechanism by which A*β* kills neurons, with some describing the activation of the extrinsic DR-mediated apoptotic pathway.^13, 33, 34, 35^ We found that 60% of the neuronal death induced by A*β* is apoptotic as cotreatment with ADDLs and the pancaspase inhibitor (Q-VD) led to a significant reduction in neuronal death compared with that induced by ADDLs alone (Figure 5a). Notice that the vehicle by itself produced some death, and that death was not reverted with Q-VD, indicating that the reduction in neuronal death in the combined treatment (ADDL+Q-VD) is due only to the prevention of apoptosis induced by ADDL. We next addressed whether FAIM-L serves as a functional antagonist of A*β*-induced neuronal death. For this purpose, we modulated the endogenous levels of FAIM-L using recombinant lentiviruses (Lv) for the overexpression (FAIM-L) or downregulation by short hairpin RNAi (shFAIM-L) (Figure 5c). In cultured neurons infected with these lentiviruses or the corresponding scrambled or empty forms, the neuronal death induced by A*β* toxicity did not show any relevant differences when FAIM-L expression was modified (Figure 5b).

### FAIM-L is required for the protective effect of TNF*α* against A*β*-induced neuronal death

**Figure 6:**
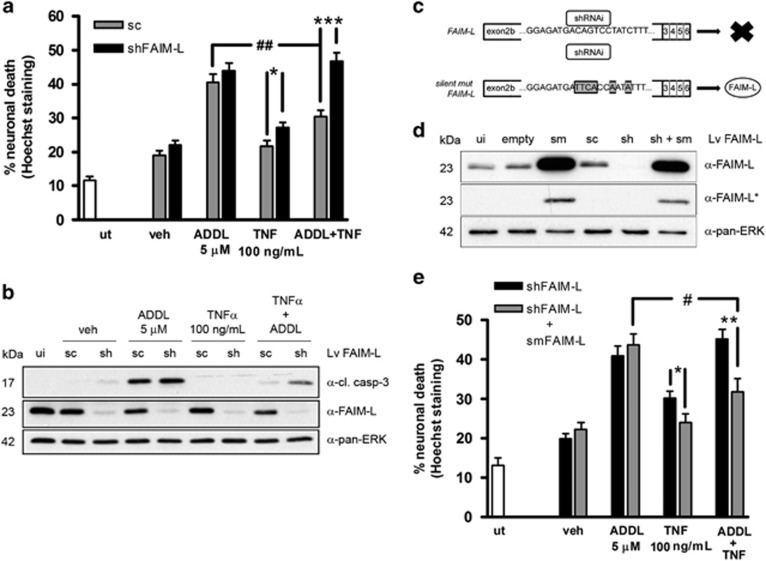
FAIM-L is necessary for TNF*α* protection against A*β*-induced death. (**a**) Neurons were infected with shFAIM-L or the corresponding scrambled (sc) control for 6 days and then treated for 48 h with 5 *μ*M ADDLs and 100 ng/ml TNF*α*. Counts of nuclei after Hoechst staining were used to determine cell death. (**b**) Primary neurons treated as in (**a**) were processed for western blot analysis of caspase-3 cleavage and FAIM-L expression. Pan-ERK was used as a loading control. (**c**) Scheme showing how the lentivirus-expressing silent mutated FAIM-L (*smFAIM-L)* avoids shRNA downregulation, giving the expression of FAIM-L. (**d**) Expression of FAIM-L after 6 days of infection with the indicated lentiviruses; *film least exposed shows that when both lentiviruses are expressed (shFAIM-L+smFAIM-L), the shFAIM-L downregulates endogenous FAIM-L. Loading control was determined with pan-ERK. (**e**) Neurons were infected as indicated and treated for 48 h with 5 *μ*M ADDLs and 100 ng/ml TNF*α*. Cell death was determined by counting nuclei after Hoechst staining. In all cases, data are mean±S.E.M. of three independent experiment, except for (**b**) that is six. One-way ANOVA with Newman–Keuls test. ***P<0.001, **P<0.01 and *P<0.05 for the same treatment; ^##^*P*<0.01 and ^#^*P*<0.05 between ADDLs and ADDLs+TNF*α*

AD brains are characterized by the presence of neuroinflammatory mediators, including increased TNF*α* during the progression of the disease.^6, 7, 8, 9^ The contribution of TNF*α* to AD pathogenesis is highly controversial. It has been described that inflammation may be protective during onset of the disease,^32^ although the persistence of inflammation may become detrimental.^36^ We have previously described that TNF*α* activates pro-survival intracellular signaling pathways through cFLIP-dependent MAPK/ERK activation.^15^ Thus, in the context of A*β* toxicity, we addressed whether TNF*α* has a protective or deleterious effect and also how this effect is regulated by FAIM-L. A relative high concentration of TNF*α* was used to reproduce the elevated levels of this cytokine in pathological conditions.^37, 38, 39^ Co-treatment of neurons with TNF*α* and A*β* resulted in a significant prevention of cell death by 47%, considering the death induced by the vehicle as basal death (Figure 6a). More importantly, the protective effect of TNF*α* was dependent on the endogenous levels of FAIM-L as FAIM-L short hairpin RNA (shRNA) blocked the protective effects of this cytokine (Figure 6a).

The cleavage of caspase-3 gives a good correlation with neuronal death, as generally it is an indicator that the caspase cascade is triggered. Caspase-3 was activated in the cultures treated with ADDLs and abrogated in neurons co-treated with TNF*α* (Figure 6b). When FAIM-L was reduced by shRNA, once again, we observed the appearance of the active caspase-3 fragment indicative of neuronal apoptosis (Figure 6b). All these results support the notion that FAIM-L is required for TNF*α* protection against ADDLs.

To ensure the specificity of the FAIM-L reduction for the results obtained regarding TNF*α* protection, we generated a lentivirus that expresses FAIM-L with a silent mutation (smFAIM-L) that cannot be recognized by shFAIM-L, thus impeding its downregulation when both lentiviruses are co-infected (Figure 6c). The smFAIM-L greatly increased the expression of FAIM-L (lane 3, Figure 6d), and this level was maintained when shFAIM-L was expressed simultaneously (lane 6, Figure 6d), in spite of shFAIM-L being highly efficient in reducing endogenous FAIM-L (lane 5, Figure 6d). In these conditions, that is, when the levels of FAIM-L were restored by smFAIM-L, TNF*α* regained its capacity to prevent the effects of A*β* toxicity in neurons (Figure 6e). On the basis of our findings, we conclude that TNF*α* protects these cells against A*β* toxicity, but that this protection occurs only when FAIM-L is present in the neuron.

### FAIM-L expression is not regulated by TNF*α*

**Figure 7:**
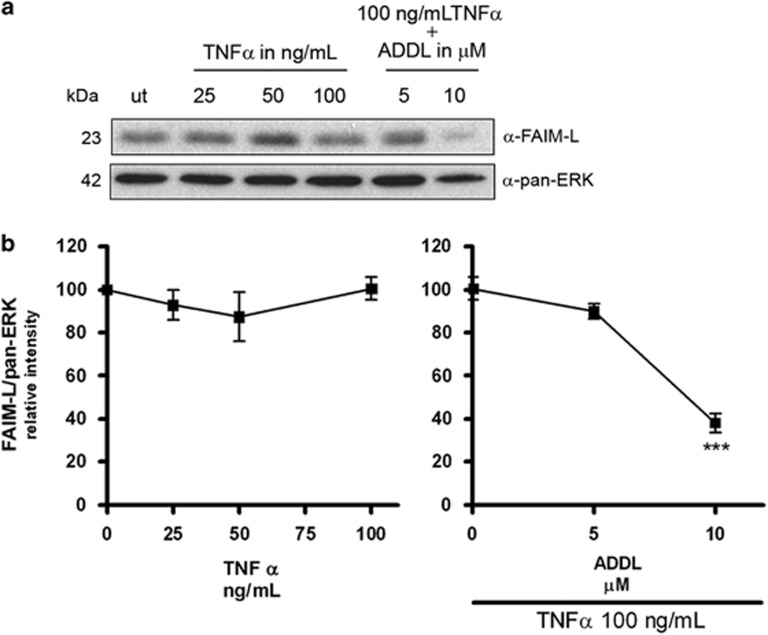
TNF*α* does not modulate the expression of FAIM-L. (**a**) Immunoblot of FAIM-L in neurons treated for 48 h with increasing concentrations of TNF*α* using pan-ERK as a loading control. (**b**) Quantification of three independent western blots (***P<0.001, *t*-test)

TNF*α* activates the transcription of protective genes by triggering the transcription factor NF*κ*B.^40, 41^ Thus, we addressed whether TNF*α* has the capacity to modulate FAIM-L expression. To this end, we treated primary cortical neurons with increasing amounts of TNF*α* and analyzed the expression of FAIM-L by western blot. TNF*α* alone did not modify the endogenous levels of FAIM-L at any of the concentrations tested. Neither in the simultaneous treatment with ADDLs was TNF*α* able to modify the decrease in endogenous FAIM-L induced by these ligands (Figures 7a and b). Accordingly, we conclude that TNF*α* does not have the capacity to reverse the downregulation of FAIM-L induced by A*β* treatment.

### Reduction of FAIM-L by A*β* suppresses the protective action of TNF*α*

**Figure 8:**
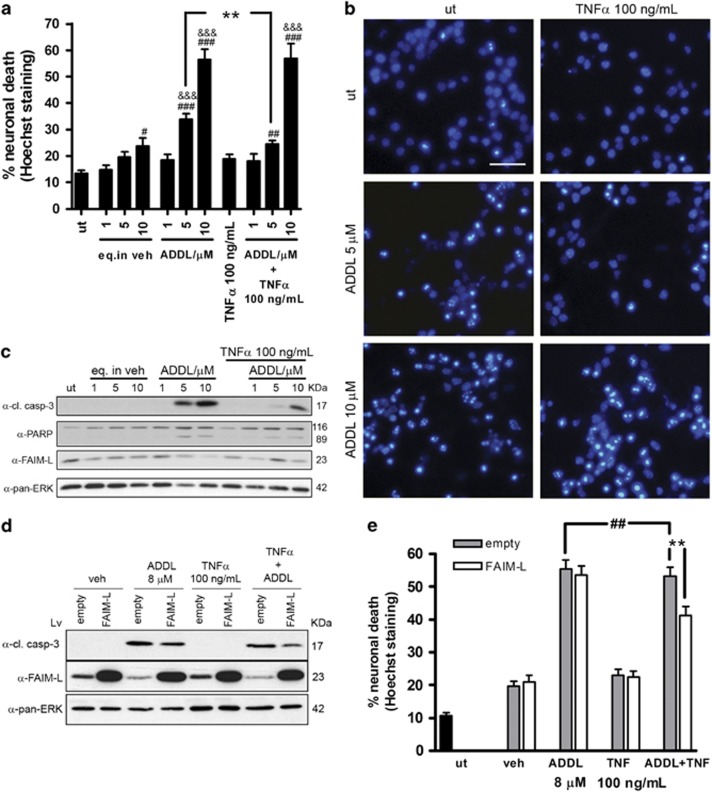
The reduction of FAIM-L by A*β* impedes TNF*α* protection. (**a**) Cortical neuronal death determined by counting nuclei stained with Hoechst after 48 h of the indicated treatments. Data are mean±S.E.M. of three independent experiments. One-way ANOVA and Newman–Keuls *post hoc*. ***P<0.001, **P<0.01 and *P<0.05 (^#^*versus* untreated; ^&^*versus* its vehicle; and * for comparisons between ADDLs and ADDLs+TNF*α*). (**b**) Representative images of nuclear Hoechst staining. Scale bar, 50 *μ*m. (**c**) Immunoblot analysis of FAIM-L, PARP, and the cleavage fragment of caspase-3 in primary cortical neurons treated as in (**a**); pan-ERK was used as a control of loading. (**d**) Neurons were infected for the overexpression of FAIM-L or an empty lentivirus for 6 days and then treated for 48 h with 8 *μ*M ADDLs and 100 ng/ml TNF*α*. Western blot analysis of caspase-3 cleavage and FAIM-L expression. Pan-ERK was used as a loading control. (**e**) Neurons were treated as in (**d**), and counts of nuclei after Hoechst staining were used to determine cell death. Graph represents mean±S.E.M. **^, ##^*P*<0.01 (*versus same treatment and ^#^between ADDLs and ADDLs+TNF*α*). One-way ANOVA followed by multiple comparison Newman–Keuls test. In all cases, the experiments were repeated three times

The results obtained hitherto show that A*β* decreases FAIM-L expression and also that this protein is required to ensure TNF*α* protection against neuronal A*β*-induced death. We further examined the capacity of TNF*α* to protect neuronal cells against A*β* toxicity induced by several amounts of A*β*. To this end, we treated cortical neurons with increasing concentrations of ADDLs in the presence or absence of TNF*α* and assessed neuronal death by counting apoptotic nuclei (Figures 8a and b). Neuronal death induced by ADDL followed a dose-dependent trend, with notable death at 5 and 10 *μ*M of A*β*. When neurons were also co-treated with TNF*α*, this cytokine significantly prevented cell death induced by 5 *μ*M of ADDLs but not by 10 *μ*M (Figures 8a and b). Western blot of caspase-3 cleavage showed a similar result; the treatment with 5 and 10 *μ*M of ADDLs activated caspase-3, but co-treatment with TNF*α* prevented this activation only in the presence of 5 *μ*M of A*β* (Figure 8c). We then sought to correlate these results with FAIM-L levels. In these conditions, as we have previously shown, there was a dose-dependent downregulation of FAIM-L expression when neurons were treated with increasing concentrations of A*β*. That is, FAIM-L expression decreased at 10 *μ*M of A*β*, but expression at 5 *μ*M was similar to that observed in vehicle-treated controls (Figure 8c). Thus, when neurons were treated with a concentration of ADDLs that reduced FAIM-L expression, TNF*α* was not able to exert a protective effect. Next, we checked whether restoring FAIM-L levels by exogenous overexpression would re-establish TNF*α*-mediated protection. To carry out this experiment we used a concentration of ADDL of 8 *μ*M because this quantity of ADDL ensured a detectable reduction in the levels of FAIM-L without significantly compromising the neuronal viability. The infection of neurons with a lentivirus that drives the exogenous overexpression of FAIM-L gave rise to a considerable increase in the levels of this protein, even when endogenous FAIM-L was reduced by treatment with 8 *μ*M of ADDLs (Figure 8d). In these conditions, TNF*α*, partially but significantly, prevented neuronal death, as assessed by caspase-3 activation and apoptotic nuclei counts (Figures 8d and e). As expected, TNF*α* did not protect neurons infected with the empty virus when they were treated with 8 *μ*M of ADDLs because in this condition the amount of FAIM-L was greatly reduced by A*β* (Figure 8e).

In conclusion, our results show that soluble A*β* downregulates the expression of FAIM-L in neurons and that this reduction impedes the TNF*α*-mediated protection against A*β*-induced neuronal death.

## Discussion

Here we show that FAIM-L levels (protein and mRNA) are reduced in patients with AD and also in the regions most affected in this disease, namely the entorhinal cortex and the hippocampus, in the transgenic mouse model of the disease (PS1xAPP). Furthermore, when we added the soluble fractions from these transgenic animals to cultured neurons, FAIM-L expression also decreased. This effect was also observed when we treated neuronal cells with ADDLs. These observations thus reveal that A*β* downregulates FAIM-L expression. We also demonstrate that the reduction in FAIM-L, achieved by shRNA or by treating the cells with A*β*, impedes TNF*α* protection against A*β*-induced cell death. All together, these findings reveal a vicious circle in which, in addition to toxicity, A*β* accumulation alters FAIM-L expression in neurons, thus switching off the protective inflammatory response induced by TNF*α*. On the basis of these results, we propose a working model whereby normal levels of FAIM-L expression in neurons allow TNF*α*-mediated protection against A*β*-induced death, thus resulting in a beneficial inflammatory response. However, when FAIM-L expression is reduced as a result of accumulation of A*β*, then TNF*α* cannot protect from A*β*, thus exposing neurons to death and contributing to neurodegeneration. Hence, the response of neurons to the same inflammatory stimulus (TNF*α*) differs in function of the levels of FAIM-L present. FAIM-L determines whether the TNF*α* response is beneficial or detrimental, therefore being critical for neuronal fate.

FAIM was first described as an antagonist of FAS.^42^ The *FAIM* gene comprises six exons, with two putative translation initiation sites within exons II and III that generate two protein isoforms.^22^ FAIM-S is widely expressed in many tissues,^22^ whereas FAIM-L is expressed exclusively in neurons.^23^ The mechanism by which FAIM-S expression is regulated in B cells has recently been described.^43^ However, the regulation of FAIM-L expression has not been resolved. We have reported on the roles of the two isoforms in the nervous system. The short form is required for neurite outgrowth through the NF*κ*B and MAPK/ERK pathways; however, FAIM-S is unable to protect neurons from death induced by trophic deprivation^44^ or DRs.^23^ Nonetheless, the neuronal long form of the protein does exert protection against TNFR1- and FAS-induced apoptosis.^23^ We recently described the molecular mechanism by which FAIM-L protects against the latter in neuronal cells.^45^ However, the mechanism by which FAIM-L mediates TNF*α* protection against A*β*-induced cell death is, at present, still unclear. In this study we show that A*β* decreases FAIM-L expression without altering the expression of FAIM-S. This observation suggests that the expression of these two forms of the FAIM protein in neurons is subjected to differential regulation. In addition, in neurons the quantity of FAIM-L is higher than the FAIM-S, as evidenced in the qPCR in Figure 4. The reduction in FAIM-L is not attributable to neuronal death, as demonstrated by the neuronal loading marker NeuN and also by the observation that at 100 *μ*g/ml the soluble fraction from 6-month-old transgenic mice caused a significant reduction in FAIM-L expression whereas the death induced was similar to that detected in age-matched WT animals. The finding that FAIM-L mRNA levels are also reduced indicates that A*β* modulates the expression of this protein rather than its degradation.

The downregulation of FAIM-L has important consequences for neuronal viability. This protein is a potent antagonist of TNF*α*- and FAS-induced death in neuronal cells. The knockdown of FAIM-L sensitizes neurons to death induced by these DRs.^23^ In this regard, it has been suggested that the expression of FAIM-L is reduced in dopaminergic neurons in Parkinson's disease, thus making this type of neurons more vulnerable to FAS-induced death.^46^ However, in general, neurons are resistant to death induced by DRs,^47, 48^ although DRs and ligands are expressed physiologically in the brain.^49, 50, 51^ Indeed, TNF*α*-induced neuronal death requires the blockage of NF*κ*B^17, 18^ or protein synthesis^19, 20^ or the depletion of FAIM-L expression.^23^ Consistent with this, as shown in Figure 6, the knockdown of FAIM-L sensitized the neurons to TNF*α*; and this effect was reverted when the cells were co-infected with smFAIM-L to restore FAIM-L expression.

In the context of AD, the function of TNF*α* is controversial. It protects neurons from A*β* toxicity^10, 11^ but also contributes to their neurodegeneration.^12, 13, 33^ Moreover its role in the aggregation of NFT has been scarcely explored.^52, 53^ In normal conditions TNF*α* acts as a modulator of brain functions,^54^ participating in the control of synaptic transmission,^55, 56^ and homeostatic plasticity also known as synaptic scaling.^57, 58, 59^ The distribution of the glial cells surrounding neuronal synapses modulate these processes.^60, 61^ The fine-tuning control of the neurotransmission mediated by TNF*α* could explain the deleterious effects on synaptic transmission when deregulated. However, the neurodegeneration observed in AD probably requires the downregulation of FAIM-L mediated by A*β*. We assessed the neuronal viability with Hoechst counts and cleavage of caspase-3. Despite that activation of this caspase has been associated with other functions different to apoptosis, in our model its cleavage appeared to be linked to neuronal death as demonstrated with the pyknotic nuclei observed with Hoechst staining, and the apparition of the 89 kDa fragment produced by the cleavage of poly (ADP-ribose) polymerase (PARP) by caspase-3. The latter is a well-defining and an ending event in the apoptotic process. Our results demonstrate that the dual role on viability of TNF*α* depends on the degree of FAIM-L expression. Although the neuronal death induced by A*β* involves mainly the activation of caspases, FAIM-L has no role on this effect. However, the expression of this protein is required for the protection conferred by TNF*α*. The overexpression of FAIM-L did not enhance this protective effect, thereby indicating that FAIM-L by itself was not responsible for the prevention of A*β*-induced death when neurons were treated with TNF*α*. Interestingly, FAIM-L does not appear to be a target gene of the TNF-induced pro-survival pathway NF*κ*B. Consequently, the treatment of neurons with TNF*α* did not reverse the downregulation of FAIM-L caused by A*β*.

Overall, our results explain why neurodegeneration is boosted by neuroinflammation when FAIM-L expression is reduced in neurons. The transgenic mouse PS1xAPP, which reproduces the human pathology and shows neuroinflammation and cortical neurodegeneration, also presents temporal and regional age-dependent vulnerability, the entorhinal cortex being the first area affected, followed by the hippocampus and subsequently an extensive part of the cortex. In the transgenic animal, both regions, the entorhinal cortex and the hippocampus, show a temporal correlation between reduced FAIM-L expression and the consequent increase in neuronal death.^29, 30, 32^ Thus, on the basis of the *in vitro* results, we propose that the increase in soluble A*β*^62, 63^ promotes the reduction in neuronal FAIM-L expression and consequently the loss of protection afforded by TNF*α* against A*β* toxicity, hence increasing neuronal death. The same hypothesis could be formulated for patients with AD. Although the progressive reduction of FAIM-L mRNA with BRAAK stage severity may mask neurodegeneration, the quantification of protein using the neuronal marker NeuN also revealed a significant decrease in FAIM-L expression between AD patients and control nondemented subjects, thus indicating that the reduction in FAIM-L is before neurodegeneration. Our observations lead us to suggest that there is a threshold of FAIM-L below which neurodegeneration is accelerated, as documented in advanced BRAAK stages,^25^ going from mild cognitive impairment (MCI) at BRAAK III–IV to AD at BRAAK V–VI. Presumably, FAIM-L is not involved in the etiology of the disease, but it can be determinant for the outcome. In this regard, we propose that the degree of FAIM-L expression or its evolution may be indicative of susceptibility to disease or disease progression.

## Materials and Methods

### Reagents

To prepare ADDLs, the soluble oligomeric forms of A*β* (oA*β*), we followed previous protocols^64, 65^ with some modifications. Cold lyophilized human sequence A*β* 1–42 (Bachem, Weil am Rhein, Germany) was resuspended in ice-cold HFIP (1,1,1,3,3,3-Hexafluor-2-propanol) (Sigma-Aldrich, Barcelona, Spain) to a final concentration of 1 mM. The solution was incubated at room temperature (r.t.) until it was transparent. Solvent was then allowed to evaporate overnight until a peptide film formed. The films were stored at *−*80ºC. The day before use, peptide films were resuspended in DMSO (Sigma-Aldrich) to a final concentration of 5 mM, and were further diluted to 100 *μ*M with DMEM/F-12 Ham's-phenol free (Life Tech, Barcelona, Spain) and incubated at 4°C for 24 h in order to achieve oligomer formation. Before use, the solution was centrifuged for 10 min at 14 000 × *g* at 4ºC. Supernatants containing the oligomers were diluted in the cell culture media to the indicated final concentrations.

Lyophilized recombinant human TNF*α* (Biotrend, Köln, Germany) was diluted to 100 mg/ml in sterile DNase/RNase-free water. A final concentration of 100 ng/ml in culture media was used.

The pan-caspase inhibitor Q-VD-OPh (MP Biomedicals, Santa Ana, CA, USA) was diluted to 20 mM in sterile DNase/RNase-free water. A final concentration of 40 *μ*M in culture media was used.

### Human samples

The study was performed using samples from 22 cases stored in the BTIN-Tissue Bank for Neurological Research, Madrid, and in the Neurological Tissue Bank of IDIBELL-Hospital of Bellvitge, Barcelona. The cases were scored for BRAAK stage for neurofibrillary tangles (0–VI). BRAAK 0 and II correspond to individuals with normal cognition and BRAAK V and VI to AD patients. There are scarcity of subjects of a similar age to AD patients without any neurofibrillary tangles (BRAAK 0), and thus we also use BRAAK II (individuals with normal cognition who present some histopathological alterations of the brain) for the statistical comparisons. Table 1 provides details of the age, BRAAK stage, gender, and delay postmortem before sample extraction.

Samples of hippocampi (60–80 *μ*g) were homogenized in 1 ml of Tripure Isolation Reagent (Roche, Sant Cugat del Vallès, Spain) as described previously for mouse samples.^29, 31, 32^ After isolation of RNA samples, RNA integrity was assessed by agarose gel electrophoresis. The yield of total RNA was determined by measuring the absorbance (260/280), and the recovery of RNA was comparable in all the groups studied (0.3–0.4 *μ*g/mg of tissue).

Retrotranscription (RT) was performed using random hexamers, 4 *μ*g of total RNA as template, and the High-Capacity cDNA Archive Kit (Applied Biosystems, Barcelona, Spain), following the manufacturer's recommendations.

### PS1_M146L_xAPP_751sl_ transgenic mouse samples

The generation of PS1_M146L_xAPP_751sl_ (PS1xAPP) mice has been reported previously.^66^ Heterozygous PS1xAPP double transgenic mice (C57BL/6 background) were generated by crossing homozygous PS1 transgenic mice with heterozygous Thy1-APP751sl ones. Only male mice were used in this study.

Laser capture microdissection of the entorhinal cortex was done as described previously.^29^ Anesthetized animals (WT and PS1xAPP at 4 and 6 months of age; *n*=4/type/age) were killed by decapitation, and brains were quickly removed and frozen. Brains were cut at 15 *μ*m on a cryostat, and sections were collected on Superfrost Plus slides (Fisher Scientific, Waltham, MA, USA) and stored at *−*80ºC. The entorhinal cortex from brain sections lightly stained with hematoxylin was identified in stereotaxic coordinates following the mouse brain atlas.^67^ Laser capture microdissection was performed using a Pix-Cell II instrument from Arcturus Engineering (Mountain View, CA, USA). Cells were captured with a 30-*μ*m laser setting and CapSure LCM Caps (Arcturus). The laser was set to a pulse of 60 mV for 50 ms. After microdissection, the capture disk was cleared of nonspecific adhering tissue using a CapSure Cleanup Pad (Arcturus). Entorhinal cortex RNA was extracted using the PicoPureTM RNA isolation kit. Capture disks with isolated material were microcentrifuged and incubated in the extraction buffer for 30 min at 42ºC. Cell extracts were then centrifuged and frozen at *−*80ºC. For RNA isolation, cell extracts were treated as indicated by the manufacturer (Arcturus). The integrity and amount of purified RNA were analyzed using the RNA 6000 Pico Assay kit (Agilent Technologies, Barcelona, Spain). Retrotranscription (RT) was done using the High-Capacity cDNA Archive Kit (Applied Biosystems), following the manufacturer's recommendations. The RT-PCR was performed basically as previously described.^29^

Soluble fractions from mouse brains at the corresponding ages were obtained as previously described.^32^ Briefly, brains were homogenated with a Teflon-glass homogenizer in cold phosphate-buffered saline (PBS) with protease inhibitors (Sigma-Aldrich) and ultracentrifuged at 120 000 × *g* at 4°C for 1 h. Immediately, samples were aliquoted and store at *−*80°C until use. Protein content was determined by means of the Lowry assay.

### Primary cortical culture

Cerebral cortices were dissected from WT fetal C57BL/6 mouse embryos at day 16 (E16). Cortices without meninges were trypsinized for 10 min at 37°C and mechanically dissociated through a Pasteur pipette and filtered through a 40-*μ*M nylon mesh in the presence of DNase I (Roche). Cells were stained with Trypan blue, counted in a Neubauer chamber, and then resuspended in Dulbecco's modified Eagle's medium (DMEM) with glutamine supplemented with 5% heat-inactivated fetal bovine serum (FBS), 5% heat-inactivated fetal horse serum (HS), 20 U/ml penicillin, and 20 *μ*g/ml streptomycin. Cells were plated in poly-L-Lysine (25 *μ*g/ml) (Sigma-Aldrich)-coated plates at a density of 1.6 × 10^5^ cells/cm^2^. Purity of the cultures was determined by counts of neurons labeled with NeuN with respect to the total number of cells labeled with Hoechst, giving 97±1.25% of neurons (*n*=3). Infections were performed just after seeding; in the case of infections with smFAIM-L, neurons were also reinfected 3 days before treatments. At 4 to 5 h after seeding, medium was replaced with serum-free Neurobasal medium supplemented with 2% B27, 1% N2, 20 U/ml penicillin, 20 *μ*g/ml streptomycin, and 0.5 mM glutaMAX (Life Tech). Culture medium was partially replaced every 3–4 days with fresh supplemented Neurobasal medium. Cell cultures were kept at 37°C in a humidified incubator with 5% CO_2_ and 95% air. Neurons were processed after 8–9 days *in vitro*.

### Cell culture

Rat pheochromocytoma PC12 cells were grown on plates coated with Type I collagen (66.4 *μ*g/ml; BD Biosciences) with DMEM supplemented with 6% heat-inactivated FBS and 6% heath-inactivated HS (Invitrogen), 10 mM HEPES, 20 U/ml penicillin, and 20 *μ*g/ml streptomycin. Cells were kept at 37°C in a humidified incubator with 5% CO_2_ and 95% air.

### Quantitative PCR

For human samples, specific gene products were amplified using the commercial hydrolysis TaqMan probes, following the instructions provided by the manufacturer (Applied Biosystems) using either an ABI Prism 7000 or 7900HT sequence detector (Applied Biosystems). A first run of amplification with three reference genes (*18S*, *GAPDH*, and *actin*) was done in order to determine equal amounts of the three genes used as standard. To perform the relative quantification using the comparative Ct delta method (Relative Quantification of Gene Expression, bulletin 2, Applied Biosystems), three independent reactions for each gene were done in parallel with the reference gene *18S*.

The qPCR in primary neuronal cultures was done using cells that were scraped out and washed once with ice-cold PBS. RNA was extracted following the protocol of the RNeasy Mini Kit (Qiagen, Barcelona, Spain). Equal amounts of RNA were converted to single-stranded cDNA using the High Capacity RNA-to-cDNA Kit (Applied Biosystems). The qPCR was performed with Sybr Green PCR Master Mix (Applied Biosystems) in an ABI Prism 7900 sequence detector (Applied Biosystems) with the conditions indicated by the manufacturer. The sequences of the primers, designed with span exon–exon junction, were as follows: *faim-l* (Mus musculus Fas apoptotic inhibitory molecule, transcript variant 1) forward 5′-GCGCGGAGCAGACTGT-3′, reverse 5′-TCCAGGCTATAGAGAGGGCTT-3′ *faim-s* (Mus musculus Fas apoptotic inhibitory molecule, transcript variant 2) forward 5′-GCGGAGCAGCCCTCTCTA-3′, reverse 5′-CCTGATGTGGTCCCATGTTCA-3′ *l27* forward 5′-AGCTGTCATCGTGAAGAA-3′, reverse 5′- CTTGGCGATCTTCTTCTTGCC-3′ and *GAPDH* forward 5′-TGTGAACGGATTTGGCCGTA-3′, reverse 5′-ACTGTGCCGTTGAATTTGCC-3′. The results of the qPCR were normalized by *l27.* Similar results were obtained with *GAPDH* as the reference gene.

### Lentiviral particles

The lentiviral particles used to modify FAIM-L quantity in neurons were: short-hairpin RNA (shFAIM-L) to knock down, FAIM-L to overexpress, and the silent mutated (smFAIM-L) to overexpress FAIM-L when the endogenous FAIM-L was downregulated by the shFAIM-L; with their corresponding scrambled (sc) form or empty vector controls.^23^ Lentiviral particles were produced as previously described.^23^

### Cell viability assays

Cell death was analyzed by nuclear Hoechst staining. At 4 h before the end of treatments, cells were stained by adding Hoechst 33258 to a final concentration of 0.3 *μ*g/ml in the media. After this time, pictures were taken in a NIKON Eclipse TE2000-s (Nikon, Tokyo, Japan). Condensed or fragmented nuclei were counted as dead cells.^68^ For each condition, 800 to 1000 cells were counted in fields of 100–200 neurons/field. All the counts were repeated at least three times in independent cultures.

### Western blot

Neurons were harvested and washed once with ice-cold PBS, then lysed in triton lysis buffer containing 50 mM Tris-HCl, pH 7.4, 150 mM NaCl, 10 mM EDTA, and 1% Triton with a cocktail of protease (Roche) and phosphatase (Sigma-Aldrich) inhibitors for 30 min at 4ºC. Supernatants were obtained after centrifugation at 14 000 × *g* for 10 min at 4ºC. Protein concentration was quantified by a modified Lowry assay (DC protein assay; Bio-Rad, Hercules, CA, USA). Next, 7–20 *μ*g of protein was resolved by SDS-polyacrylamide gels and transferred onto PVDF (Millipore Ibérica, Madrid, Spain). Membranes were incubated with blocking solution (5% non-fat dry milk in TBS with 0.1% tween-20 (TBS-T)) for 1 h at r.t. Primary antibodies prepared in blocking solution were incubated overnight at 4ºC. After washes with TBS-T, membranes were probed with the appropriate specific peroxidase-conjugated secondary antibody and developed using the EZ-ECL chemiluminescence detection kit (Biological Industries, Kibbutz Beit Haemek, Israel). The primary antibodies used were anti-FAIM-L (antibody generated with the specific sequence presented in FAIM-L) and anti-FAIM-S (both in-house, tested and validated for both WB and immunohisto/cytochemistry^23^); anti-caspase-3, anti-PARP (Cell Signalling Technologies, Beverly, MA, USA); anti-NeuN, anti-panERK (Millipore); and anti-tubulin (Sigma-Aldrich).

### Animal ethics committees

All experiments involving the PS1xAPP mice were carried out in accordance with the European Union regulations (Council Directive 86/609/ECC of 24 November 1986) and approved by the Committee of Animal Use for Research at Malaga University, Spain (RD 1201/2005 of 10 October 2005). Regarding the primary cultures, the procedures followed the experimental protocol approved by the Vall d'Hebron Institutional Review Board (CEIC).

### Statistical analysis

All the experiments were performed at least three times. The statistics used are indicated in the figure legends with the significance obtained. For the analysis of human samples, we used nonparametric tests: the equivalent to ANOVA test Kruskal–Wallis followed by Dunn's multiple comparison test; and the nonparametric *t*-test Mann–Whitney. For the statistical analysis of the *in vitro* experiments, we used parametric tests: one-way ANOVA with multiple Newman–Keuls test to analyze the differences between groups and *t*-test to compare two sets of data.

## Figures and Tables

**Table 1:** Human samples

**Mean age±S.D.**	**Sex**	**Delay post-mortem mean h±S.D.**	**BRAAK**
47.83±5.81	4 Male and 2 female	7.2±3.9	0
72.66±9.43	4 Male and 2 female	6.2±4.5	II
85.75±4.42	1 Male and 3 female	11.2±4.0	V
77.16±13.24	2 Male and 4 female	11.0±5.8	VI

## Abbreviations

A*β*: amyloid-*β*
AD: Alzheimer's disease
ADDL: A*β*-derived diffusible ligand
DR: death receptor
FAIM: Fas apoptotic inhibitory molecule
FAIM-L: long form of FAIM protein
FAIM-S: short form of FAIM protein
FLIP-L: FLICE-like inhibitory protein, long isoform
Lv: lentivirus
MAPK/ERK: mitogen-activated protein kinase/extracellular-signal-regulated kinase
MCI: mild cognitive impairment
NFT: neurofibrillary tangles
NF*κ*B: nuclear factor-*κ*B
oA*β*: oligomeric A*β*
PARP: poly (ADP-ribose) polymerase
PS1_M146L_xAPP_751sl_: Presenilin 1 with mutation M146L x human APP751 (Amyloid Precursor Protein) carrying the London (V717I) and the Swedish (K670N/M671L) mutations
qPCR: quantitative PCR
RNAi: RNA interference
sc: scrambled
shRNA: short hairpin RNA
shFAIM-L: short hairpin RNA for FAIM-L
smFAIM-L: lentivirus that expresses FAIM-L with a silent mutation
SOM/NPY: somatostatin/neuropeptide Y
TNF*α*: tumor necrosis factor-*α*
TNFR1: TNF receptor 1 also called TNFRSF1A
WT: wild type
