# Supplementary material for: Amyloid-β reduces the expression of neuronal FAIM-L, thereby shifting the inflammatory response mediated by TNFα from neuronal protection to death
Source: Cell Death Dis. 2015 Feb 12;6(2):e1639–. doi: 10.1038/cddis.2015.6 (PMC4669818; doi:10.1038/cddis.2015.6)
Supplement: Supplementary Information [file cddis20156x1.doc]

**SUPPLEMENTARY INFORMATION**

**MATERIALS AND METHODS**

**Immunohistochemistry**

After deep anesthesia with sodium pentobarbital (60 mg/kg), 2- and 6-month-old PS1xAPP and WT mice were perfused transcardially with 0.1 M phosphate-buffered saline (PBS), pH 7.4 followed by 4% paraformaldehyde, 75 mM lysine, and 10 mM sodium metaperiodate in 0.1 M phosphate buffer (PB), pH 7.4. Brains were then removed, post-fixed overnight in the same fixative solution at 4°C, cryoprotected in 30% sucrose, and sectioned at 40 μm thickness in the coronal plane on a freezing microtome. Free-floating sections were first treated with 3% H_2_0_2_/3% methanol in PBS and with the avidin-biotin Blocking Kit (Vector Labs, Sant Cugat del Vallès, Spain), and then incubated overnight with rabbit polyclonal anti-FAIM-L antibody (1:2000 dilution) for 24 h at 4ºC. Biotinylated secondary antibody (1:500 dilution; Vector Labs) was used to detect tissue-bound primary antibody, followed by streptavidin-conjugated horseradish peroxidase (1:2000; Sigma-Aldrich). Peroxidase reaction was visualized with 0.05% 3-3’-diaminobenzidine tetrahydrochloride (DAB) (Sigma-Aldrich), 0.03% nickel ammonium sulphate, and 0.01% hydrogen peroxide in PBS. Sections were then mounted in gelatin-coated slides, air-dried, dehydrated in graded ethanols, cleared in xylene, and coverslipped with DPX (BDH) mounting medium. The specificity of the immune reaction was achieved by omitting primary antibody. Sections from PS1xAPP and WT mice were assayed simultaneously for immunohistochemistry using the same batches of solutions to minimize variability in immunolabeling conditions. Quantifications were performed with ImageJ software. Integrated density measurement represents the sum of the gray values of all of the pixels in the selection divided by the number of pixels.

**SUPPLEMENTAL FIGURE LEGENS**

**SUPPLEMENTAL FIGURE 1. mRNA of proteins involved in TNF signaling and DR antagonists in human hippocampal samples.**

**(a)** Quantitative PCR of proteins involved in TNF signaling in post-mortem hippocampal samples. As others, we found that TNFα (TNF) mRNA increases with the progression of Alzheimer’s disease (AD). In relation to TNF receptors, there is a significant increase in TNFR1 (TNFRSF1A) between BRAAK 0 and BRAAK V; however this increase is not maintained in BRAAK VI. No significant differences were observed for TNFR2 (TNFRSF1B). Regarding the proteins involved in the regulation of TNF signaling, mRNA for cIAP1 (BIRC2) and SODD (BAG4) is reduced, with no significant changes for cIAP2 (BIRC3) or A20 (TNFAIP3) mRNA. The latter increases but does not reach significance. **(b)** mRNA levels of the DR antagonists cFLIP (CFLAR) and Lifeguard (FAIM-2). No differences were observed in the amount of cFLIP mRNA in the BRAAK stages analyzed. The mRNA of Lifeguard is reduced in AD patients. Data are mean ± SD of three independent experiments. Kruskal-Wallis test followed by Dunn's multiple comparison test. *** p<0.0001; ** p<0.001 and * p<0.005.
