# Supplementary figures and images for: Amyloid-β reduces the expression of neuronal FAIM-L, thereby shifting the inflammatory response mediated by TNFα from neuronal protection to death
Source: Cell Death Dis. 2015 Feb 12;6(2):e1639–. doi: 10.1038/cddis.2015.6 (PMC4669818; doi:10.1038/cddis.2015.6)

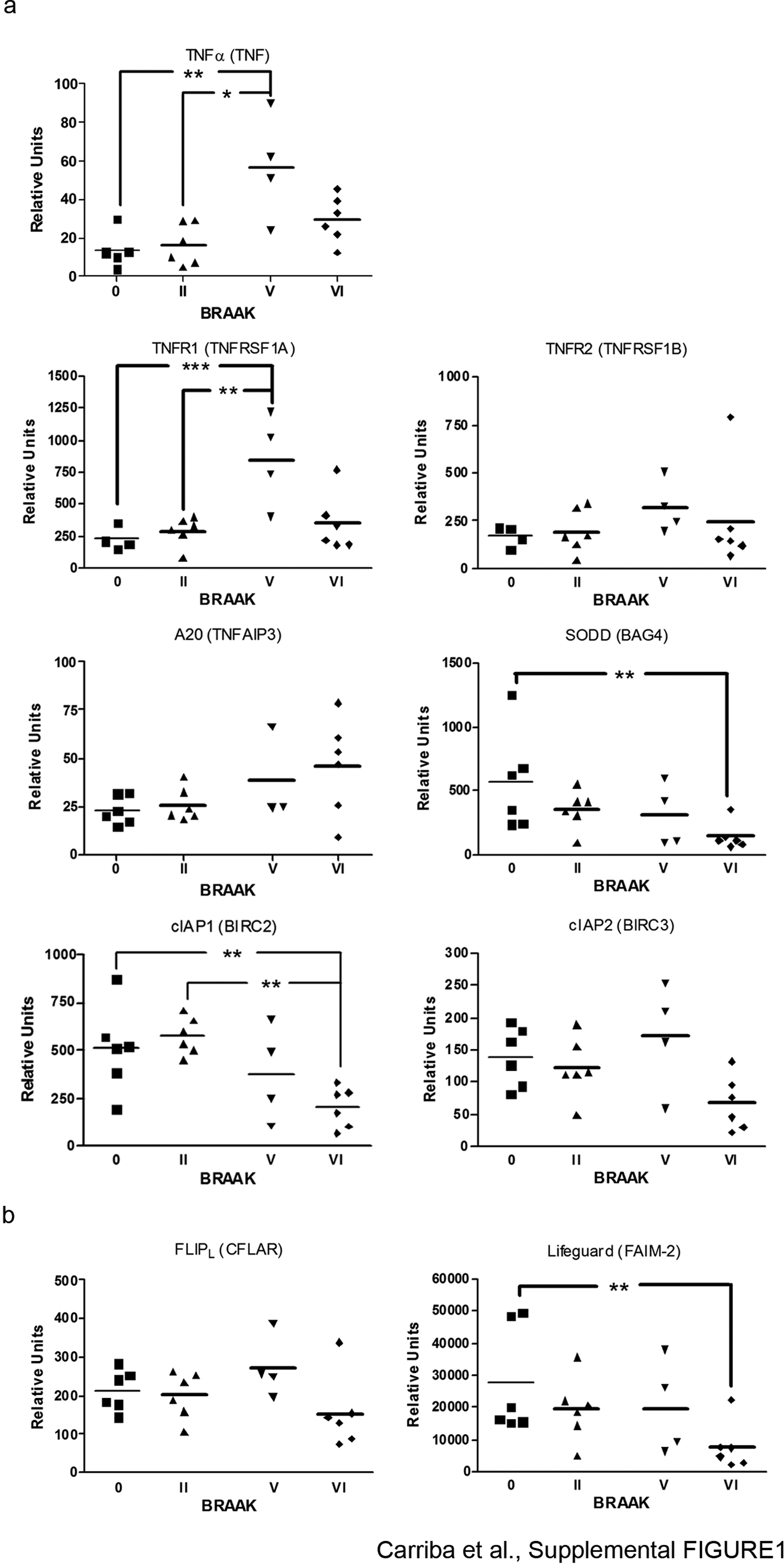

Supplement: Supplementary Figure 1 [file cddis20156x2.tif]
